# Editorial: Integrated Motor-Cognitive Aerobic Rehabilitation Approaches in Parkinson's Disease

**DOI:** 10.3389/fneur.2021.677721

**Published:** 2021-08-04

**Authors:** Margherita Canesi, Maria Felice Ghilardi

**Affiliations:** ^1^Department of Parkinson's Disease, Movement Disorders and Brain Injury Rehabilitation, Moriggia Pelascini Hospital, Gravedona, Italy; ^2^School of Medicine, City University of New York, New York, NY, United States

**Keywords:** rehabilitation, neuroplasticity, biomarkers, Parkinson' s disease, neuroprotection

Parkinson's Disease (PD) is the second most common neurodegenerative disease, involving chronic and disabling movement disorder with prevalence increasing with age (1). Over the past three decades, the number of people with PD has exceeded 6 million and this number is now projected to double by 2040 (2).

The causes of this disease are still largely unknown. PD is likely the consequence of the combined effects of multiple risk factors, including genetics and environment (3). The typical motor signs and symptoms of PD, i.e., bradykinesia, rigidity, resting tremor, postural instability, result from rearrangements within the cortico-basal ganglia-thalamo-cortical loops following the degeneration of nigro-striatal dopaminergic neurons. As a consequence of striatal dopamine deficiency, cognition is called to compensate for motor automaticity deficits such as gait disturbances, and thus reducing the capacity for dual tasking (4).

PD is nevertheless characterized by an extensive range of non-motor features, such as hyposmia, sleep disorders, depression, anxiety, changes in cognitive abilities, and pain. These symptoms, which may be already present in the prodromal stages of PD or may emerge later on in the early and advanced phases of the disease, are due to the central role played by the basal ganglia. Indeed, these nuclei participate in multiple cortico-subcortical re-entrant pathways involving motor, associative and limbic functions that account for most of the non-motor symptoms (5).

Another important characteristic of PD is the impairment of long-term potentiation phenomena that, together with sleep disorders present in this disease, decrease the capacity for plasticity in PD (6). Both these aspects have important repercussions on the formation and retention of new memories and skills (6) and thus on the effects of rehabilitation treatments.

PD treatment is symptomatic since no cure exists. Levodopa (the “gold standard”) is an effective and well-tolerated dopamine replacement agent (DRT). However, levodopa therapy is complicated by motor response fluctuations and dyskinesias that are experienced by up to 90% of patients after about 10 years of DRT (7). In addition, most of the patients develop also non-motor fluctuations with a further deterioration in terms of autonomy and quality of life (8). These problems are likely expressions of maladaptive plasticity phenomena induced by chronic DRT (9).

An increasing number of studies show that exercise improves both motor and non-motor problems as well as the secondary complications of immobility (10). Furthermore, exercise can have a disease-modifying influence through neuroplasticity mechanisms such as synaptogenesis, neurogenesis, neuronal sprouting, and synaptic strength. Rehabilitation based on goal-directed motor skill learning along with cognitive involvement (including feedback, cueing, dual tasking, motivation) leads to improvement in motor performance (11). Interestingly, aerobic exercise improves motor as well as cognitive performance based on attention and executive functions in PD with long-lasting effects (6, 12–15). The bases of the benefits of aerobic exercise are not yet clarified but they may include improvement of plasticity-related phenomena, with the recruitment of immune system resources (16), increasing blood flow and trophic factor signaling (17) as well as sleep improvements (18).

Van Wegen et al. designed an original study with a single case experimental design to explore the possible changes of motor and non-motor performances as well as of biomarkers for neuroplasticity (BDNF) and neurodegeneration (NfL and alfasynuclein) in response to intensive physical training. The control group will perform continuous aerobic exercise. The intensive exercise strategy adopted is the “high-intensity interval training” characterized by several intervals of short lasting and high intensity bouts alternated with low intensity bouts. The study design consents the collection of individual response patterns to different exercises strategies for motor, non-motor, and biomarkers. The single subject research methodology allows the gathering of new data with small samples.

Daily life activities require the ability to perform simultaneously two or more goal-direct tasks, for example employing cognitive resources while walking to handle complex environmental conditions. In PD, dual tasking is impaired compared to age-matched healthy controls due to the decrease of automaticity and to the increased reliance on cognition to control gait. Treadmill training may then improve dual tasking during gait through its cognitive and motor effects.

D'Cruz et al. in a “single session proof-of-concept study” ascertained whether split-belt training produces greater effects than tied-belt training on dual-task walking and turning performance in parkinsonian patients with freezing of gait and age-matched normal controls. The results revealed that split-belt training improved dual-task walking and turning more than tied-belt training in both subject groups. The better performance after split-belt training is likely due to the reduced cognitive expenses required for the gait control. Noteworthy, advantages from the split-belt training were larger just post-training as well as when tested the day after.

Future studies will determine whether these effects can last for a longer time with a promising impact on freezing of gait and the risk of fall in daily life.

One of the most common triggers of freezing gait in PD is the experience troublesome turning in place leading to a higher risk of falling and a decrease in quality of life. To date, the cueing strategy used for turning provides external timing (auditory) and no goal-directed movements are available. Janssen et al. assessed augmented reality visual cues in patients with PD and freezing of gait. Contrary to the authors' expectations, augmented reality visual cues worsened freezing of gait, as well as measures of axial kinematic, turn scaling, and timing. These results may be because goal-directed turning might be insufficient and augmented reality might represent a dual-task strategy. Moreover, the smart glasses used in this training might be too heavy and patients might not adapt to either augmented or virtual reality.

Rhythmic auditory stimulation with gait rehabilitation can be a useful strategy in PD, as auditory cueing can compensate for the loss of automatic and rhythmic movements with a reshaping of the connectivity within the fronto-centroparietal and temporal network. The results of the pilot study by Naro et al. indicated that RAS-assisted treadmill gait rehabilitation within conventional physiotherapy programs induces motor performance improvement that is greater in parkinsonian patients that have deep brain stimulation implants. Therefore, deep brain stimulation may enhance the beneficial effects of rhythmic auditory stimulation with gait rehabilitation by improving plasticity.

Music-assisted treadmill training (MATT) is a rehabilitation strategy combining treadmill training, rhythmic auditory cueing, and visual feedback. Gooßes et al. presented the results of a feasibility pilot randomized controlled trial study with MATT in patients with PD with and without DBS. In particular, the authors assessed the patients' study protocol acceptance, the study protocol transferability into clinical routine, the adverse events, and the training perception. MATT demonstrated to be feasible, safe, and satisfying for patients with and without DBS. In this trial, dropout rates were high, primarily in the experimental group, but this occurred in the recruitment phase. The authors suggest that the analysis of the dropout cohort could be used to explain patients' adherence and expectation.

The PAIRED study proposed by Hackney et al. is a randomized, controlled trial in mild-moderate PD to compare the effect of partnered dance aerobic exercise vs. a walking aerobic exercise in a long-term intervention (16 months) for off-time, cognition, and neuroprotection. The authors used neuromelanin loss and iron accumulation as biomarkers of neuroprotection measured using neuromelanin-sensitive MRI and iron-sensitive MRI sequences. The expected outcomes are reduction in off-time, improvement of visuospatial cognition, and slower neurodegeneration in the partnered dance aerobic exercise group compared to the walking aerobic exercise group.

Altogether, the evidence provided by these studies suggests that the neuroplasticity needed to overtake the altered basal-ganglia circuitry can be facilitated by a synergic use of goal-directed practice exercises and aerobic training.

## Author Contributions

MC draft the paper. MG revised it critically. Both authors contributed to the article and approved the submitted version.

## Conflict of Interest

The authors declare that the research was conducted in the absence of any commercial or financial relationships that could be construed as a potential conflict of interest.

## Publisher's Note

All claims expressed in this article are solely those of the authors and do not necessarily represent those of their affiliated organizations, or those of the publisher, the editors and the reviewers. Any product that may be evaluated in this article, or claim that may be made by its manufacturer, is not guaranteed or endorsed by the publisher.
